# Granular cell ameloblastoma: A rare case report with public health implications

**DOI:** 10.1097/MD.0000000000041992

**Published:** 2025-05-09

**Authors:** Shyamkumar Sriram, Shamimul Hasan, Mambakkam Jayakanth, Syed Ansar Ahmad, Anoop Kumar Narayanan

**Affiliations:** a Department of Rehabilitation and Health Services, College of Health and Public Service, University of North Texas, Denton, TX; b Department of Oral Medicine and Radiology, Faculty of Dentistry, Jamia Millia Islamia, New Delhi, India; c Department of Internal Medicine, Patiala Heart Institute, Patiala, India; d Department of Oral and Maxillofacial Surgery, Faculty of Dentistry, Jamia Millia Islamia, New Delhi, India; e School of Family Health Studies, Kerala University of Health Sciences, Kozhikode, Kerala.

**Keywords:** ameloblastoma, case report, granular cell ameloblastoma, multilocular radiolucency, odontogenic tumors

## Abstract

**Rationale:**

Ameloblastomas are benign odontogenic tumors that exhibit local aggressiveness and a high potential for recurrence. Their histopathological diversity and potential to cause significant anatomical and functional complications often make diagnosis and treatment challenging. This case highlights the clinical, radiographic, and histopathological features of a rare granular cell ameloblastoma and underscores the importance of a radical surgical approach to management.

**Patient concerns:**

A 46-year-old female was referred to the Outpatient Department with a complaint of swelling on the right side of her face for 10 months. The swelling started as a small, asymptomatic enlargement of the lower jaw and gradually increased over the past several months.

**Diagnoses:**

The clinical examination revealed noticeable facial asymmetry with a diffuse, firm to bony-hard swelling in the right mandibular region. Intraorally, a lobulated, bony-hard swelling with significant cortical expansion in the right lower jaw was observed. The mandibular occlusal radiograph showed a “soap bubble” appearance with multilocular radiolucency, cortical plate thinning, and disruption. The orthopantogram displayed a well-defined multilocular radiolucent lesion with root resorption, displaced teeth, and a “tooth floating in air” appearance. An incisional biopsy revealed tumor islands or follicles of odontogenic epithelium. Tall columnar ameloblast-like cells were arranged in a palisaded fashion at the periphery, and stellate reticulum-like cells were at the center. Large granular cells containing eosinophilic cytoplasmic granules confirmed the diagnosis of granular cell ameloblastoma. The patient was diagnosed with granular cell ameloblastoma.

**Interventions:**

A lower cheek flap was raised using a Roux lip split incision, followed by segmental mandibulectomy and resection of the tumor mass. The resected specimen’s histopathological findings were consistent with the incisional biopsy.

**Outcomes:**

The patient exhibited uneventful postoperative recovery with no signs of recurrence or metastasis during a 2-year follow-up period.

**Lessons:**

Granular cell ameloblastoma, although uncommon, demands careful differentiation from other odontogenic neoplasms due to its distinctive histological characteristics. An integrated approach, combining clinical, radiographic, and histopathological assessments, is essential for precise diagnosis and optimal treatment planning. Given the tumor’s locally aggressive nature, radical surgical treatment is often necessary to prevent recurrence. Long-term follow-up is vital to monitor for potential recurrence and ensure complete disease control.

## 1. Introduction

The World Health Organization describes ameloblastoma as a benign epithelial intraosseous odontogenic tumor, marked by its progressive growth, potential to destroy adjacent tissues, and propensity for local recurrence if not completely excised. The first documented case of ameloblastoma was reported by Cusack in 1827, and Ivy and Churchill gave the condition its name in 1930.^[1–4]^

Ameloblastoma is a benign, locally aggressive epithelial tumor arising from enamel, dental follicles, periodontal ligaments, or the linings of odontogenic cysts.^[5,6]^ Robinson defined the tumor as “usually unicentric, nonfunctional, intermittent in growth, anatomically benign and clinically persistent.”^[7,8]^

The annual incidence of ameloblastoma is estimated to be 0.5 cases per million people. They are the second most common odontogenic tumor, constituting 1% of all oral tumors and around 9% to 11% of odontogenic tumors.^[6,9]^

Although the exact cause of ameloblastoma remains unclear, it may be associated with localized trauma, nutritional deficiency, inflammation, mutations, and/or molecular aberrations affecting various signaling pathways.^[10,11]^ Moreover, genetic and molecular aberrations may contribute to the aggressiveness and metastatic potential of the tumor.^[7,11,12]^

Ameloblastomas are more commonly seen in certain age groups and locations, with approximately 80% of cases occurring in the mandibular body and ramus region, predominantly in individuals aged 30 to 50. Nevertheless, the lesion can affect individuals of all age groups, including children.^[13–15]^ The tumor is typically asymptomatic, slow-growing, and locally aggressive, characterized by cortical expansion, perforation, and infiltration into nearby soft tissues.^[15,16]^ In some cases, ameloblastoma is an incidental radiographic finding.^[17,18]^

The orthopantomogram serves as a valuable first-line diagnostic tool, and reveals well-defined, expansile, unilocular or multilocular radiolucencies with a characteristic “soap bubble” appearance. The extent of the tumor and cortical bone destruction can be assessed using computed tomography.^[6]^

Ameloblastomas show diverse microscopic patterns, with the granular cell pattern being uncommon, representing about 3% to 5% of all histologic variants of ameloblastomas.^[19–22]^

Granular cell ameloblastoma (GCA) is distinguished by the presence of granular cells at the center of the follicles, which progressively replace the stellate reticulum. These cells represent a transitional phase in the tumor’s lifecycle, starting from stellate cells, and evolving into granular cells that produce granules, ultimately leading to cyst formation.^[9,23,24]^

Krompecher (1918) was the first to recognize granular cell changes in the histopathology of ameloblastoma, referring to them as pseudoxanthomatous cells.^[25–28]^ Although earlier regarded as a marker of aging or degenerative changes in chronic lesions, this variant has also been identified in young individuals and clinically aggressive lesions. The tumor has an aggressive course, and an increased risk of recurrence and metastasis.^[13,15,26–29]^

Given the rarity of the GCA subtype and its potential to be mistaken for other odontogenic and non-odontogenic lesions with a granular cell component, it is crucial to have a clear understanding of the distinctive features of this locally aggressive neoplasm.^[19]^

Considering its aggressive nature and high recurrence rates, the primary treatment approach is complete excision, accompanied by adequate reconstruction.^[8,30]^ The preferred treatment for large multicystic GCA is segmental resection, followed by reconstruction of the resulting defect. Recent guidelines recommend a minimum margin of 1 to 2 cm for segmental resections.^[31]^

Here, a rare case of GCA of the mandible is reported. Due to its high recurrence rate, this subtype needs to be distinguished from other variants of ameloblastoma and granular cell lesions.

## 2. Case report

A 46-year-old female was referred to the Outpatient Department with a complaint of swelling on the right side of her face for 10 months.

### 2.1. History and clinical examination

The swelling, which started as a small, asymptomatic enlargement of the lower jaw, gradually increased over the past several months. The patient had no history of trismus, pain, pus discharge, paresthesia, or difficulty in chewing or speaking. She mentioned having a right mandibular molar extraction a year ago. Her medical and family histories were unremarkable. The patient has not underwent any treatment before.

**Figure 1.**
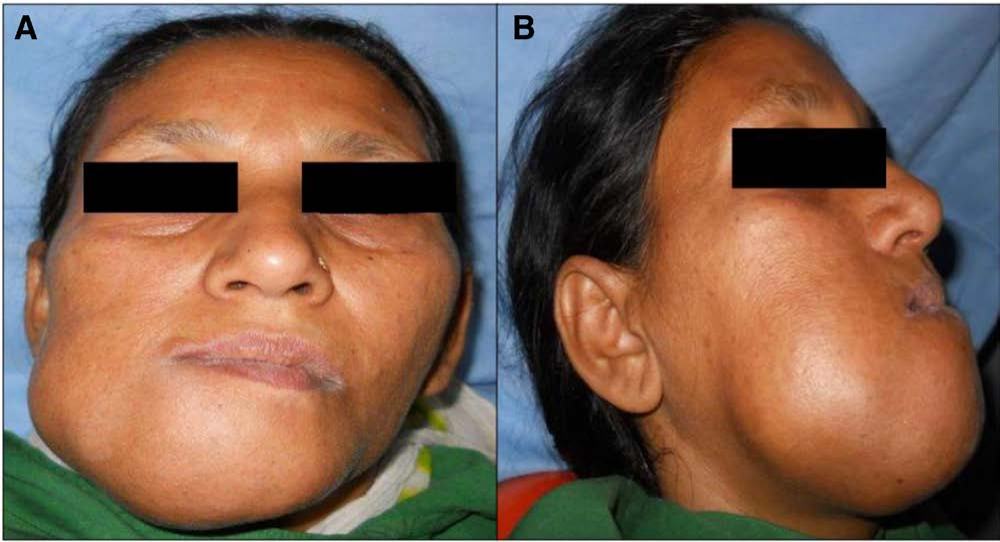
(A and B): Frontal and lateral view of the patient showing the extraoral extent of the lesion.

On extraoral examination, a marked facial asymmetry, with a diffuse, smooth-surfaced swelling on the right side of the face, affecting the mandibular body and ramus was observed. The swelling measured about 6 × 6 cm, extending superiorly up to the ala-tragus line, inferiorly 3 cm below the inferior border of the mandible, and anteroposteriorly from the left parasymphyseal region to the right angle of the mandible. Nasolabial fold obliteration with deviation of the lip to the left side were also noted. The skin over the swelling appeared shiny and non-pinchable, with no signs of ulceration, discharge, sinus tract, or other changes. On palpation, the swelling was firm to bony hard and non-tender, with no increase in surface temperature. No associated lymphadenopathy was present (Fig. 1A and B).

**Figure 2.**
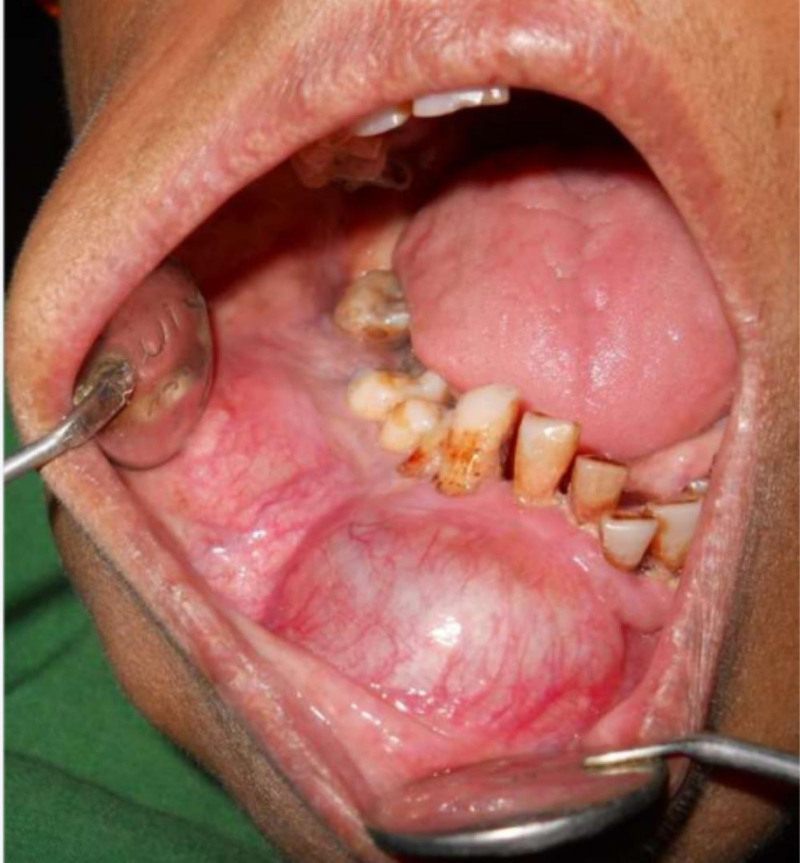
Intra-oral swelling in the right mandibular body with vestibular obliteration.

Intra-oral examination revealed a diffuse, bony-hard, lobulated swelling with significant cortical expansion in the right lower jaw, extending from the left mandibular anterior teeth to the right retromolar region. The swelling completely obliterated the buccal vestibule. The affected teeth were non-tender, displaced, and demonstrated grade I mobility. The overlying mucosa appeared shiny and stretched. Palpatory findings were suggestive of a bony hard & non-tender swelling (Fig. 2).

### 2.2. Differential diagnosis

Considering the history and clinical presentation, the lesion was differentially diagnosed as an odontogenic neoplasm (Ameloblastoma, odontogenic myxoma, keratocystic odontogenic tumor), malignancy within odontogenic neoplasms (Ameloblastic carcinoma/sarcoma), central giant cell granuloma and vascular neoplasm of bone (Hemangioma).

### 2.3. Investiagtions

**Figure 3.**
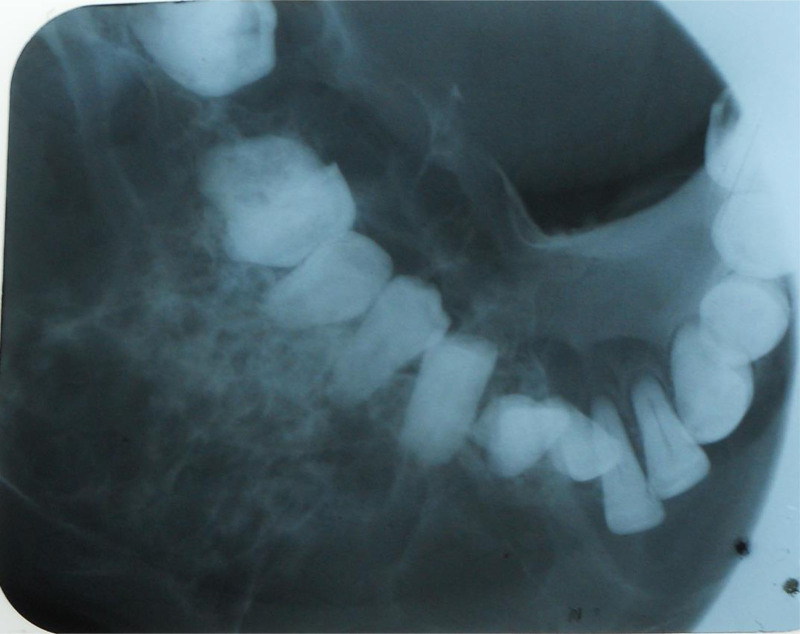
Occlusal radiograph showing a multilocular radiolucent lesion with cortical expansion and tooth displacement.

The mandibular occlusal radiograph showed a characteristic “soap bubble” multilocular radiolucency, affecting the marrow bone and leading to uneven expansion, with more pronounced buccal expansion than lingual. Thinning and disruption of the buccal and lingual cortex, with adjacent teeth displacement was also seen (Fig. 3).

**Figure 4.**
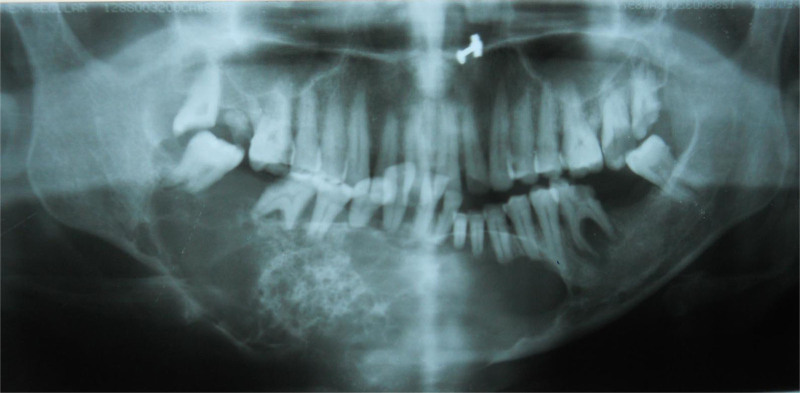
OPG showing a multilocular radiolucent lesion with “tooth floating in air” appearance. OPG = orthopantomogram.

The orthopantogram demonstrated a well-demarcated multilocular radiolucency extending from the right mandibular ramus to the left mandibular second premolar region. Irregular bony trabeculae within the lesion indicated the persistence of bone after destruction. Root resorption and interdental bone loss were observed in the affected teeth, resulting in a “tooth floating in air” appearance (Fig. 4). Cone-beam computed tomography was advised to assess the lesion’s extent, three-dimensional (3D) location, and proximity to anatomic landmarks, but the patient declined the procedure due to financial constraints.

**Figure 5.**
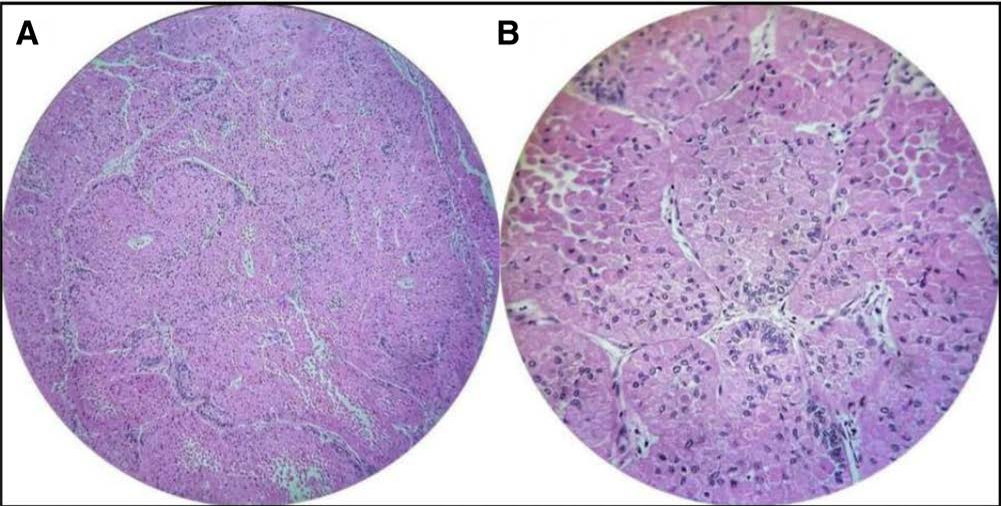
(A) Photomicrograph showing tumor islands with peripheral tall columnar cells and central granular cells (H & E 10×). (B) Photomicrograph showing ameloblastomatous follicles with peripheral tall columnar cells and central cells showing prominent eosinophilic granules (H & E 40×).

Routine hematology and biochemical tests were within the normal range. An incisional biopsy was performed following the patient’s informed consent. Hematoxylin and eosin (H&E) staining revealed odontogenic epithelium as tumor islands and follicles within the connective tissue stroma. Tall columnar ameloblast-like cells arranged in a palisaded fashion were observed at the periphery of the follicles, and stellate reticulum-like cells at the center. Large granular cells, containing abundant eosinophilic granules in their cytoplasm occupied the center of the tumor follicles (Fig. 5A and B). The histopathological features confirmed the diagnosis of granular cell ameloblastoma.

### 2.4. Treatment

A lower cheek flap was raised using a Roux lip-split incision, followed by segmental mandibulectomy and tumor resection. The resected specimen’s histopathological findings were consistent with the incisional biopsy.

### 2.5. Outcome and follow up

Post-operative recovery was uneventful, with no clinical or radiographic signs of recurrence or metastases during a two-year follow-up. Informed consent was obtained from the patient for publication of this case report.

## 3. Discussion

Ameloblastoma is a locally invasive tumor originating from odontogenic epithelium. It consists of proliferating enamel organ-like tissue that has not differentiated into hard tissue and lacks enamel and dentin. Various sources have been proposed for the epithelium of origin in ameloblastoma, including the epithelial lining of odontogenic cysts, the dental lamina or enamel organ, disruptions in the developing enamel organ, basal cells of the surface epithelium, and heterotopic epithelium from other body parts.^[9,28]^

The World Health Organization (2017) classifies ameloblastomas into 4 types: conventional ameloblastoma (formerly termed “solid/multicystic ameloblastoma”), which includes follicular, plexiform, acanthomatous, desmoplastic, granular cell, and basal cell variants, either singly or in combination; unicystic ameloblastoma, with luminal, intraluminal, and mural variants; extraosseous/peripheral ameloblastoma; and malignant or metastatic ameloblastoma.^[1,4,32]^

The peak incidence of ameloblastoma differs by type and age: the conventional type is most common in the 4th and 5th decades, the unicystic type in the 2nd and 3rd decades, and the extraosseous/peripheral type in the 5th to 7th decades.^[33]^

Ameloblastoma usually presents with minimal signs and symptoms. In certain instances, radiological changes may be accidentally identified on radiographs taken for other purposes.^[18]^ The most common presenting symptom of ameloblastoma is a slow-growing, asymptomatic jaw swelling. Other clinical signs include soft tissue infiltration, mobility of adjacent teeth, and dental malocclusion. Pain is an uncommon symptom, typically arising from hemorrhage within or near the tumor, or due to neural invasion.^[17,34]^ Ameloblastoma typically grows in the buccolingual direction, causing notable expansion. On average, ameloblastomas measure around 4 cm at the time of presentation.^[17,34–36]^

The age, clinical features, and tumor location in our case align with the published literature.

Granular cell ameloblastoma (GCA) displays the typical radiographic features of ameloblastoma, including multilocularity and a tendency for cortical expansion and perforation. The radiographic appearance is depicted as a “soap bubble” when the loculations are large and “honeycombed” when they are smaller, depending on their size. Root resorption of adjoining teeth is frequently observed.^[13,15,17]^

In the reported case, a multilocular radiolucency was observed in the posterior mandible, accompanied by cortical expansion and adjoining teeth root resorption.

The follicular (32.5%) and plexiform (28.2%) subtypes are the most common histological types, followed by the acanthomatous (12.1%) and desmoplastic (4–13%) variants. The granular cell (3–5%) and basal cell (2%) ameloblastomas are less frequently observed subtypes.^[12,37,38]^

GCA is distinguished by the transition of stellate reticulum-like cells into granular cells with dense eosinophilic cytoplasm. These granular cells have copious cytoplasm with eosinophilic granules resembling lysosomes, detectable through ultrastructural and histochemical analysis.^[15,19,27,29,38,39]^

The rarity of GCAs is highlighted in the literature. In a clinicopathological study, Kameyama et al^[40]^ reported that only 1 out of 77 ameloblastoma cases was of the granular cell type. Reichart et al^[37]^ reviewed literature from 1960 to 1993 and documented only 56 cases of GCA out of 1593 cases with available histologic data.

Several theories have been proposed to explain the nature of the granules in GCA. These include the possibility that the granular cell change may result from a degenerative process in a chronic lesion, dysfunctional neoplastic cells, a metabolic response to aging, or serve as a marker of a more aggressive disease progression.^[29,37,41]^ Nevertheless, it is still uncertain whether the granular cell change in ameloblastoma is a result of degeneration or an indication of a more aggressive disease.^[41,42]^

Ultrastructural and histochemical studies indicate that granular cells are lysosomes, with their accumulation in the cytoplasm possibly caused by impaired lysosomal enzyme function or lysosome-associated proteins, leading to the buildup of substrates typically degraded in the endosome-lysosome system.^[9,21,22,39]^

Granularity in cells may be attributed to aging or degeneration. It is proposed that lysosomes aggregate as their ability to eliminate unwanted components diminishes with age.^[39,43]^ Additionally, the increased expression of fibronectin, a biomarker of replicative senescence, in granular cells suggests that these changes may be associated with age-related alterations.^[29,39,44]^

The aging phenomenon is further illustrated by the fact that patients with granular cell ameloblastomas are, on average, 8 years older than those with conventional ameloblastomas. Furthermore, granular cell ameloblastomas exhibit a longer average symptom duration of 15.3 years compared to 2.3 to 5.8 years in conventional ameloblastomas.^[36,37,44]^ However, the aging phenomenon is no longer regarded as the cause of granularity, as this variant has also been observed in younger patients.^[29]^

Published literature suggests that granularity in GCAs may result from increased apoptosis and subsequent phagocytosis by adjoining neoplastic cells, driven by the expression of death signaling molecules.^[9,21,39,43]^ Immunohistochemical studies have demonstrated an increase in apoptotic cells and a reduction in the expression of antiapoptotic factors like Bcl-2 and p53 proteins in GCA.^[15,21,27,29]^ Published literature shows that signaling molecules such as Wnt-5a and β-catenin are produced in greater amounts in granular cells, but the impaired transport or secretion leads to their aggregation as autophagosomes.^[21,43]^

Oral lesions with similar histomorphology, including granular cell tumor, granular cell odontogenic tumor, granular cell myoblastoma, granular cell ameloblastic fibroma, and congenital epulis, are considered in the differential diagnosis of GCA. Although these tumors share similar granular cell morphology, they differ in their histogenesis. Immunohistochemistry may serve as a diagnostic aid in differentiating GCA from these tumors.^[19–22,36,37,39]^

The management of ameloblastomas should be guided by the patient’s history, clinical and radiographic assessments, and histopathological features.^[45]^

Granular cell ameloblastomas managed with conservative approaches such as enucleation or curettage are more likely for recurrence, as the tumor’s border within the cancellous bone extends beyond the visible gross specimen and radiographic boundaries of the lesion. As a result, radical surgical approaches, precise preoperative diagnosis, and long-term follow-up are advised.^[9,14,29]^

GCAs carry a higher risk of recurrence (33.3% more likely than other types)^[37]^ and can metastasize to distant organs and tissues, including the lungs and thoracic vertebrae.^[28,38]^ The tumor’s aggressive nature may be linked to its higher recurrence rates and metastatic potential.^[21,38]^

In this case, the aggressive nature of the lesion was evident by the destruction of a large portion of the mandibular body and root resorption of adjacent teeth. The patient had undergone a right mandibular molar extraction one year before the diagnosis of GCA. However, it is unclear whether the extraction triggered the lesion or if it was present inherently before the extraction and developed independently.

## 4. Conclusion

Granular cell ameloblastoma (GCA) is a rare condition with distinct histopathological features. Due to its higher recurrence rate, aggressive nature, and need for long-term follow-up, it must be differentiated from other subtypes. Understanding the composition of the granules in GCA and similar lesions is crucial for accurate diagnosis and comprehension of its clinical behavior. A clear understanding of GCA’s clinicopathological features can improve diagnostic accuracy, reduce recurrence rates, and enhance treatment outcomes.

## 5. Recommendations

- Granular cell ameloblastoma, a rare variant of ameloblastoma, requires careful management due to its unique histological features and potential for aggressive behavior.
- Surgical excision is typically the primary treatment approach, with an emphasis on achieving clear margins to minimize recurrence risk. Long-term follow-up is essential to monitor for any signs of recurrence.
- Multidisciplinary collaboration among oral surgeons, pathologists, and oncologists can enhance patient outcomes by providing comprehensive care tailored to individual needs.

## 6. Patient perspectives

The patient experienced uneventful healing after the segmental mandibulectomy and tumor resection, and was completely satisfied with the treatment protocol. Post-operative recovery was uneventful, with no clinical or radiographic signs of recurrence or metastases during a two-year follow-up.

## Author contributions

**Conceptualization:** Shyamkumar Sriram, Shamimul Hasan.

**Investigation:** Shamimul Hasan, Syed Ansar Ahmad, Anoop Kumar Narayanan.

**Methodology:** Shyamkumar Sriram, Shamimul Hasan, Mambakkam Jayakanth.

**Resources:** Mambakkam Jayakanth.

**Supervision:** Shamimul Hasan.

**Validation:** Syed Ansar Ahmad, Anoop Kumar Narayanan.

**Visualization:** Syed Ansar Ahmad.

**Writing – original draft:** Shyamkumar Sriram, Shamimul Hasan, Mambakkam Jayakanth.

**Writing – review & editing:** Shamimul Hasan.

## References

[1] Speight PM Takata T. New tumour entities in the 4th edition of the World Health Organization Classification of Head and Neck tumours: odontogenic and maxillofacial bone tumours. Virchows Arch. 2018;472:331–9. 28674741 10.1007/s00428-017-2182-3 PMC5886999

[2] You Z Liu SP Du J Wu YH Zhang SZ. Advancements in MAPK signaling pathways and MAPK-targeted therapies for ameloblastoma: a review. J Oral Pathol Med. 2019;48:201–5. 30489659 10.1111/jop.12807

[3] Aramanadka C Kamath AT Kudva A. Recurrent ameloblastoma: a surgical challenge. Case Rep Dent. 2018;2018:8271205. 29682361 10.1155/2018/8271205 PMC5841118

[4] Jurado-Castaneda E Ramirez-Martinez CM Alonso-Moctezuma A. Conventional ameloblastoma. A case report with microarray and bioinformatic analysis. Diagnostics (Basel). 2022;12:3190. 36553196 10.3390/diagnostics12123190 PMC9777305

[5] Mendenhall WM Werning JW Fernandes R Malyapa RS Mendenhall NP. Ameloblastoma. Am J Clin Oncol. 2007;30:645–8. 18091060 10.1097/COC.0b013e3181573e59

[6] Adeel M Rajput MSA Arain AA Baloch M Khan M. Ameloblastoma: management and outcome. Cureus. 2018;10:e3437. 30546984 10.7759/cureus.3437 PMC6289562

[7] Ruslin M Hendra FN Vojdani A. The epidemiology, treatment, and complication of ameloblastoma in east-indonesia: 6 years retrospective study. Med Oral Patol Oral Cir Bucal. 2018;23:e54–8. 29274152 10.4317/medoral.22185 PMC5822540

[8] Ashwinirani SR Das R Pawar K. Granular cell ameloblastoma: a case report of a rare histological entity with the review of literature. Med J Dr. D.Y. Patil Vidyapeeth. 2023;16(Suppl 2):S307–9.

[9] Masthan KM Anitha N Krupaa J Manikkam S. Ameloblastoma. J Pharm Bioallied Sci. 2015;7:S167–70. 26015700 10.4103/0975-7406.155891 PMC4439660

[10] Hendra FN Van Cann EM Helder MN. Global incidence and profile of ameloblastoma: a systematic review and meta-analysis. Oral Dis. 2020;26:12–21. 30614154 10.1111/odi.13031

[11] Ragunathan YT Kumar SK Janardhanam D Ravi A Santhanam V Ramdas MN. Prevalence and epidemiological profile of ameloblastoma in India: a systematic review and meta-analyses. Asian Pac J Cancer Prev. 2022;23:3601–10. 36444570 10.31557/APJCP.2022.23.11.3601 PMC9930951

[12] Kajla P Lata J Aggarwal S. A combination of follicular and plexiform ameloblastoma: a rare case report. Natl J Maxillofac Surg. 2022;13:S212–5. 36393941 10.4103/njms.NJMS_82_16 PMC9651216

[13] Chavhan A Pakhale A Patil S Hande A Tehzeeb H Akolkar S. Ameloblastoma with the hybrid desmoplastic and plexiform pattern: a case report. Cureus. 2024;16:e61686. 38975386 10.7759/cureus.61686 PMC11226227

[14] Arora S Mujhib A Diwakar G Amberker V. Granular cell ameloblastoma: a case report with a brief note on review of literature. Egypt J Ear Nose Throat Allied Sci. 2014;15:267–9.

[15] Mathew A. A case report on granular cell ameloblastoma - a rare histological entity. Indian J Radiol Imaging. 2020;30:225–8. 33100694 10.4103/ijri.IJRI_145_19 PMC7546283

[16] Vohra FA Hussain M Mudassir MS. Ameloblastomas and their management: a review. Pak J Surg(Int). 2009;14:136–42.

[17] Ghai S. Ameloblastoma: an updated narrative review of an enigmatic tumor. Cureus. 2022;14:e27734. 36127985 10.7759/cureus.27734 PMC9481193

[18] Kreppel M Zoller J. Ameloblastoma-clinical, radiological, and therapeutic findings. Oral Dis. 2018;24:63–6. 29480593 10.1111/odi.12702

[19] Nikitakis NG Tzerbos F Triantafyllou K Papadimas C Sklavounou A. Granular cell ameloblastoma: an unusual histological subtype report and review of literature. J Oral Maxillofac Res. 2011;1:e3. 24421980 10.5037/jomr.2010.1403 PMC3886069

[20] Sukumaran R Somanathan T Sen A. Granular cell ameloblastoma: a rare and unique variant. Indian J Case Rep. 2022;8:360–2.

[21] Taneeru S Guttikonda VR Yeluri S Madala J. Granular cell ameloblastoma of jaw – report of a case with an emphasis on its characterization. J Clin Exp Dent. 2013;5:e154–6. 24455072 10.4317/jced.51015 PMC3892250

[22] Martin Y Sathyakumar M Premkumar J Magesh KT. Granular cell ameloblastoma. J Oral Maxillofac Pathol. 2017;21:183. 28479717 10.4103/jomfp.JOMFP_45_15 PMC5406811

[23] Kumar M Iyer VK Mathur S Barwad A. Granular cell ameloblastoma-diagnosis on aspiration cytology. Diagn Cytopathol. 2019;47:1120–2. 31433575 10.1002/dc.24306

[24] Lee SK Kim YS. Current concepts and occurrence of epithelial odontogenic tumors: I. Ameloblastoma and adenomatoid odontogenic tumor. Korean J Pathol. 2013;47:191–202. 23837011 10.4132/KoreanJPathol.2013.47.3.191 PMC3701814

[25] Krompecher E. The histogenesis and morphology of adamantinomas and other jaw tumors. Beitr Pathol Anat. 1918;64:165–97.

[26] Kulkarni D Ingale Y Ingale M Ajabrao BN Mayank M Kulkarni A. Granular cell ameloblastoma: a rare case report and review of literature. Indian J Dent Res. 2018;29:830–5. 30589015 10.4103/ijdr.IJDR_407_17

[27] Hunasgi S Koneru A Chauhan DS Guruprasad Y. Rare giant granular cell ameloblastoma: a case report and an immunohistochemical study. Case Rep Dent. 2013;2013:372781. 23533826 10.1155/2013/372781 PMC3606760

[28] Bansal A Bhatnagar A Saxena S. Metastasizing granular cell ameloblastoma. J Oral Maxillofac Pathol. 2012;16:122–4. 22434948 10.4103/0973-029X.92988 PMC3303505

[29] Yamunadevi A Madhushankari GS Selvamani M Basandi PS Yoithapprabhunath TR Ganapathy N. Granularity in granular cell ameloblastoma. J Pharm Bioallied Sci. 2014;6(Suppl 1):S16–20. 25210361 10.4103/0975-7406.137253 PMC4157257

[30] Hoyos AM Teshima THN Dias CD Pinto CAL Coutinho-Camillo CM Lourenço SV. Granular cell ameloblastoma: retrospective clinical and histopathological findings of case series and review of literature. Austin J Dent. 2018;5:1118.

[31] Razmi SE Hayden RE Chang BA. Large mandibular mass with several floating teeth: granular cell ameloblastoma. J Surg Case Rep. 2023;2023:rjad666. 38111489 10.1093/jscr/rjad666 PMC10725788

[32] Morice A Neiva C Fabre M. Conservative management is effective in unicystic ameloblastoma occurring from the neonatal period: a case report and a literature review. Oral Surg Oral Med Oral Pathol Oral Radiol. 2020;129:e234–42. 31562035 10.1016/j.oooo.2019.08.009

[33] Wright JM Vered M. Update from the 4th Edition of the World Health Organization classification of head and neck tumours: odontogenic and maxillofacial bone tumors. Head Neck Pathol. 2017;11:68–77. 28247226 10.1007/s12105-017-0794-1 PMC5340735

[34] Urechescu H Banu A Baderca F. Ameloblastoma of the mandible in a 16-year-old female-case report. Medicina (Kaunas). 2023;60:66. 38256328 10.3390/medicina60010066 PMC10819258

[35] Boffano P Cavarra F Tricarico G. The epidemiology and management of ameloblastomas: a European multicenter study. J Craniomaxillofac Surg. 2021;49:1107–12. 34583885 10.1016/j.jcms.2021.09.007

[36] Hartman KS. Granular-cell ameloblastoma. Oral Surg Oral Med Oral Pathol. 1974;38:241–53. 4528580 10.1016/0030-4220(74)90063-2

[37] Reichart PA Philipsen HP Sonner S. Ameloblastoma: biological profile of 3677 cases. Eur J Cancer B Oral Oncol. 1995;31B:86–99. 7633291 10.1016/0964-1955(94)00037-5

[38] Rattanakuntee S Thosaporn W Ketchaikosol N Imerb N. Granular cell ameloblastoma in maxilla: a report of rare case. Oral and Maxillofacial Surgery Cases. 2023;9:100299.

[39] Dave A Arora M Shetty VP Saluja P. Granular cells in ameloblastoma: an enigma in diagnosis. Indian J Dent. 2015;6:211–4. 26752883 10.4103/0975-962X.165048 PMC4691993

[40] Kameyama Y Takehana S Mizohata M. A clinicopathological study of ameloblastomas. Int J Oral Maxillofac Surg. 1987;16:706–12. 3125270 10.1016/s0901-5027(87)80057-7

[41] Cadavid AMH Teshima THN Pinto CAL Camillo CMC Lourenço SV. Ameloblastoma with distinctive granular cell pattern: an 8 case study. Autops Case Rep. 2018;8:e2018052. 30775327 10.4322/acr.2018.052 PMC6360828

[42] Thillaikarasi R Balaji J Gupta B. Cystic granular cell ameloblastoma. J Maxillofac Oral Surg. 2010;9:310–3. 22190813 10.1007/s12663-010-0083-y PMC3177435

[43] Gupta S Grewal H Sah K. Granular cell ameloblastoma showing desmoplasia. Ann Saudi Med. 2012;32:537–40. 22871627 10.5144/0256-4947.2012.30.5.1342 PMC6081002

[44] Lapthanasupkul P Poomsawat S Chindasombatjaroen J. Investigation of basement membrane proteins in a case of granular cell ameloblastoma. Int J Oral Sci. 2012;4:45–9. 22361945 10.1038/ijos.2012.9 PMC3421479

[45] Jahanshahi G Arzhang E Derisavy S Davoodi L Shakeri S. Granular cell type of ameloblastoma. Dent Res J (Isfahan). 2018;15:224–7. 29922343 PMC5958541

